# Editorial: Antiviral drugs: neurotoxicity and neurodevelopmental effects

**DOI:** 10.3389/fnmol.2024.1526357

**Published:** 2024-11-26

**Authors:** Isabella Zanella, Daniela Zizioli, Eugenia Quiros-Roldan, Aditya N. Bade

**Affiliations:** ^1^Department of Molecular and Translational Medicine, University of Brescia, Brescia, Italy; ^2^Medical Genetics Laboratory, Diagnostic Department, ASST Spedali Civili di Brescia, Brescia, Italy; ^3^Division of Infectious and Tropical Diseases, ASST Spedali Civili di Brescia, Brescia, Italy; ^4^Department of Clinical and Experimental Sciences, University of Brescia, Brescia, Italy; ^5^Department of Pharmacology and Experimental Neuroscience, University of Nebraska Medical Center, Omaha, NE, United States

**Keywords:** anti-viral drugs, combination anti-retroviral therapy (cART), neurotoxicity, neurodevelopmental toxicity, neuropsychiatric adverse events

Several anti-viral drugs like those used to treat human immunodeficiency virus (HIV) infections have been associated with neurotoxicity or neurodevelopmental impairments following *in utero* exposures. The aim of this Research Topic was to provide an updated overview on this Research Topic. The published articles explored the argument using classical *in vitro* and animal models, and new emerging tools like cerebral organoids, covering several aspects of the proposed Research Topic.

Monnerie et al. explored the possibility that elvitegravir (EGV), belonging to the integrase strand transfer inhibitor (INSTI) class of antiretroviral drugs, may contribute to white matter damage in people living with HIV. The authors used mature rat cerebral cortical oligodendrocyte cultures as an *in vitro* model and found that EVG reduced oligodendrocyte differentiation, particularly at later stages. They investigated the involved mechanisms and found that EVG modulated the expression of sterol regulatory element-binding protein-1 and –2 (SREBP-1 and SREBP-2), major regulators of cellular lipid metabolism. Their modulation was accompanied by reduced expression of key enzymes involved in fatty acid synthesis, while cholesterol pathways seemed unaffected. In contrast, the INSTI raltegravir (RAL) had no effect on SREBP expression. Unexpectedly, the addition of palmitate, a fatty acid, to oligodendrocyte cultures did not prevent EVG-induced inhibition of oligodendrocyte differentiation, while addition of serum albumin did prevent these effects.

Rodent models are often used to evaluate neuronal toxicity of antiretroviral drugs or HIV-associated insults, however experimental observations may not consistently coincide with results from human neuronal models. To overcome this problem, Starr et al. established an *in vitro* model of human induced pluripotent stem cell (iPSC)-derived cortical glutamatergic neurons. The authors first characterized their model, then assessed the effects of EVG and RAL exposure. The authors previously demonstrated that EVG but not RAL was neurotoxic in primary rat neuroglial cultures and that its toxicity was driven by the activation of the integrated stress response (ISR; Stern et al., 2018). Here, they did not observe toxicity with either drug, measured by microtubule-associated protein 2 (MAP2) staining. However, electrophysiological measures suggested sub-lethal damage. RNA expression analysis further revealed that both INSTIs downregulated genes involved in extracellular matrix interaction, cell motility and morphogenesis.

LaNoce et al. described the use of human iPSC-based forebrain organoids as models of human early cortical development to explore the effect of the INSTIs dolutegravir (DTG) and RAL. Previously, this model was successfully used to study the effects of Zika virus on neurodevelopment (Qian et al., 2016). Researchers treated early-stage cortical organoids with DTG or RAL within the range of cord blood concentrations. DTG exposure resulted in reduced organoid size and density of neural rosette structures and increased caspase-3 staining, while RAL had no appreciable effects. The authors performed RNA expression analyses on treated organoids and found that DTG exposure resulted in the upregulation of cellular stress, response to glucose starvation and amino acid stimulus pathways and downregulation of pathways involved in neuron differentiation and migration, CNS development and neuroblast proliferation. Immuno-histological analysis revealed reduced neurogenesis with fewer neural stem cells and postmitotic neurons. Further, they demonstrated that DTG induced ISR activation with the upregulation of downstream effectors, which may disrupt cell cycle progression and impair neurogenesis.

A three-dimensional stem cell-derived brain organoid developmental model was also employed by Caiaffa et al. to analyze the effect of DTG with or without folic acid (FA) exposure in the earliest maturation stages. RNA expression analysis revealed that DTG exposure modulated the expression of genes involved in neurodevelopment, cell cycle, extracellular matrix, and membrane and motor proteins, as well as inducing the expression of the folate receptor FOLR1. FA exposure in the presence of DTG attenuated the alteration of gene expression, suggesting a protective role of FA. Using multimodal optical instruments, the authors found that organoids exposed to DTG presented biomechanical alterations like higher surface stiffness, lower internal stiffness and decreased volume during maturation, that were not rescued or mitigated by FA supplementation.

Foster et al. evaluated safety and benefits of injectable long-acting DTG nano-formulation (NDTG) on fetal neurodevelopment compared to daily oral administration of native DTG in pregnant mice. The authors found that intramuscular injection of NDTG achieved therapeutic plasma DTG concentrations in pregnant dams. These plasma drug concentrations were comparable to levels observed in dams following daily oral DTG administration. Of note, NDTG injections resulted in significantly lower levels of DTG biodistribution to the embryo brain compared to daily oral DTG administration. T_1_ mapping evaluations using *in utero* magnetic resonance imaging (MRI) indicated that lower drug exposure to the embryo brain using injectable NDTG can prevent native drug induced oxidative stress. Further, non-targeted proteomic profiling of whole embryo brain tissues from the oral native DTG group showed oxidative stress, mitochondrial dysfunction, reduced energy production, cell damage and impaired neuronal or synaptic development. These drug-induced adverse effects on embryo brain were prevented or attenuated when NDTG was administered, signifying that delivery of DTG as injectable long acting nanoformulations (NDTG) can prevent DTG-associated neurodevelopmental impairments and improve safety of DTG during pregnancy.

Finally, Dhume et al. explored the long-term effects of perinatal anti-retroviral therapy (ART) exposure on neurocognitive and motor performance in a murine model. Pregnant mice were exposed to a combination of ritonavir-boosted atazanavir (ATV/r), belonging to the class of the HIV protease inhibitors (PIs), with either an abacavir/lamivudine (ABC/3TC) or tenofovir/emtricitabine (TDF/FTC) nucleoside reverse transcriptase inhibitor (NRTI) backbone. Following *in utero* exposure, the authors found that both regimens were associated with long-term adverse neurodevelopmental outcomes in progeny observed in adulthood, but different motor and cognitive deficits were observed, dependent on the NRTI backbone. Exposure to the regimen containing TDF/FTC was associated with increased locomotor activity and exploratory behavior and reduced anxiety, while exposure to the ABC/3TC containing regimen induced greater anxiety. Working and social memory were impaired with both regimens. The researchers then showed that the ABC/3TC + ATV/r regimen led to brain volumetric reduction and reduced hippocampal neuronal counts, correlating with memory deficits. Further, altered mRNA expression of hippocampal neurotransmitter receptors as well as brain-derived neurotrophic factor (BDNF) and its receptors was observed in both ART regimens. Sex differences were also observed in behavioral testing and gene expression evaluations highlighting the importance of including sex as a factor in analyses.

In conclusion, the research papers collected in this Research Topic presented the most advanced studies on the neurotoxic and neurodevelopmental effects of anti-viral drugs. Many questions are still open, but the new emerging tools described in this Research Topic may lead to new knowledge in the field.
